# Woven endobridge device compared to stent-assisted coiling and microsurgical clipping for intracranial aneurysms: A systematic review and meta-analysis

**DOI:** 10.1097/MD.0000000000050462

**Published:** 2026-08-28

**Authors:** Adil Ahmed, Ocílio Ribeiro Gonçalves, Paweł Łajczak, Kenzo Ogasawara Donato, Maria Antonia Oliveira Machado Pereira, Filipe Virgilio Ribeiro, Anderson Matheus Pereira da Silva, Jose Fernando Olvera-Castro, João Vitor Andrade Fernandes, Muhammad Nawaz Khan, Syed Sarmad Bukhari

**Affiliations:** a Department of Medicine, Northwest School of Medicine, Peshawar, Pakistan; b Department of Medicine, Federal University of Piauí, Teresina, Brazil; c Department of Medicine, Medical University of Silesia, Katowice, Poland; d Department of Medicine, Bahiana School of Medicine and Public Health, Salvador, State of Bahia, Brazil; e Department of Medicine, State University of Piauí, Teresina, Brazil; f Faculty of Medicine, Barão de Mauá University Center, Ribeirão Preto, SP, Brazil; g Department of Physiology and Pharmacology, Federal University of Pernambuco, Recife, PE, Brazil; h Facultad de Ciencias de la Salud, Universidad Autónoma de Baja California, Tijuana, México; i Medical Science Center, Federal University of Paraíba, João Pessoa, PB, Brazil; j Department of Neurosurgery, Lady Reading Hospital, Peshawar, Pakistan; k Neurosurgical Service, Beth Israel Deaconess Medical Center, Harvard Medical School, Boston, USA.

**Keywords:** intracranial aneurysm, meta-analysis, microsurgical clipping, stent-assisted coiling, wide-neck aneurysm, Woven EndoBridge (WEB)

## Abstract

**Background:**

Endovascular techniques and devices, including Woven EndoBridge (WEB) and stent-assisted coiling (SAC), expand the choice of therapies for complex aneurysms, while the evidence directly comparing them to microsurgical clipping (MSC) remains very limited. This meta-analysis compares the safety and efficacy of WEB vs SAC and MSC in patients with ruptured and unruptured intracranial aneurysms.

**Methods:**

This study followed PRISMA guidelines. A comprehensive search of PubMed, Embase, and Scopus identified comparative cohort studies of WEB vs SAC and MSC for wide-neck intracranial aneurysms. Outcomes included occlusion, favorable functional/neurological outcome, defined as a modified Rankin Scale (mRS) score of 0–2, complications, and mortality. Statistical analyses were performed in R Studio. Heterogeneity was assessed with I^2^ and leave-one-out (LOO) sensitivity, and publication bias with funnel plots and Egger’s test.

**Results:**

Seventeen observational studies with a total of 2289 patients were included. When compared with mechanical circulatory support (MSC), the WEB device was associated with lower rates of adequate occlusion (RR 0.77; 95% CI 0.69–0.86; *P* < .001) and higher rates of incomplete occlusion (RR 4.61; 95% CI 2.12–10.01; *P* < .001). No significant differences were found in mortality or favorable neurological outcomes (defined as modified Rankin Scale [mRS] 0–2). In comparisons with stent-assisted coiling (SAC), the WEB device showed similar rates of complete occlusion (RR 0.98; *P* = .617), retreatment (RR 0.79; *P* = .508), and thromboembolic complications (RR 0.59; *P* = .137). The WEB device did demonstrate a significantly lower incidence of procedure-related complications (RR 0.49; 95% CI 0.29–0.85; *P* = .011).

**Conclusion:**

MSC offers the most durable occlusion, whereas WEB and SAC are less invasive, with WEB showing better safety than SAC but lower durability than MSC, supporting individualized, evidence-based treatment.

## 1. Introduction

Significant advances have occurred in the treatment of cerebral aneurysms over the past three decades,^[1]^ expanding the spectrum of lesions amenable to endovascular management.^[2]^ Endovascular therapy is a continuously developing field, with ongoing improvements in techniques and devices. In this context, devices such as flow diverters, Woven EndoBridge (WEB), and stent-assisted coiling (SAC) have been recently introduced into clinical practice.^[3,4]^

SAC extends treatment options to aneurysms with complex morphology and has been associated with occlusion rates superior to standalone coiling, though at the cost of a heightened risk of ischemic stroke.^[5]^ The WEB device, in clinical use since 2011, has become a well-established intrasaccular flow-disruption technique, particularly suited to wide-neck bifurcation aneurysms, and carries the additional benefit of obviating the need for prolonged antiplatelet therapy.^[6]^

Nonetheless, aneurysm recurrence following WEB embolization has been documented in approximately 10% of cases, typically necessitating repeat intervention.^[7]^ By comparison, microsurgical clipping (MSC) achieves immediate aneurysm obliteration and is generally associated with lower recurrence rates.^[8,9]^ Despite this, direct comparative evidence evaluating WEB, SAC, and microsurgical clipping head-to-head remains confined to a small number of studies.^[10,11]^

To address this gap, we conducted the first systematic review and meta-analysis to evaluate the efficacy and safety of the WEB device compared with SAC and MSC for treating ruptured and unruptured intracranial aneurysms. This study aims to provide comprehensive evidence to inform clinical decision-making and identify areas that require further investigation.

## 2. Methods

This systematic review and its accompanying meta-analysis were registered a priori with PROSPERO (registration number CRD420251150114). All procedures adhered to the PRISMA 2020 reporting framework and were guided by the methodological standards set out in the Cochrane Handbook for Systematic Reviews of Interventions.^[12]^

As this study is a systematic review and meta-analysis of previously published, de-identified aggregate data, approval by an institutional review board or ethics committee, and individual patient informed consent, were not required.

### 2.1. Eligibility criteria

We included comparative studies directly evaluating the Woven EndoBridge (WEB) device versus stent-assisted coiling (SAC) and microsurgical clipping (MSC) for the endovascular treatment of wide-neck intracranial aneurysms, whether ruptured or unruptured. Eligible studies met the following criteria:

Patient Population: Adult patients (aged > 18 years) presenting with an unruptured or ruptured wide-neck aneurysm (neck width ≥ 4 mm or dome-to-neck ratio < 2).
Intervention: Endovascular or microsurgical treatment with embolization using a WEB device of any generation and any diameter.
Comparators: Stent-assisted coiling (SAC) with any type of stent, and microsurgical clipping (MSC).
Outcomes: the reporting of at least one outcome: Adequate or complete aneurysm occlusion at follow-up (using Raymond-Roy classification or other similar classification system); Hemorrhagic complication related to external ventricular drain; Ischemic or hemorrhagic complications related to procedures; Need for retreatment or recurrence; Good functional outcome (modified Rankin Scale 0–2) at follow-up; Procedural and/or fluoroscopy time. Both prospective and retrospective observational cohort studies that provided adjusted and/or unadjusted outcomes, and that either did not use PSM, or that reported adjusted measures after using PSM, were eligible for inclusion. Single arm studies, case series, reviews, commentaries, articles indexed for congress but without full paper, and articles not reporting one or more of the defined outcome measures were eligible for exclusion.

### 2.2. Search strategy, study selection, and data extraction

A comprehensive literature search was performed across PubMed, Embase, and Scopus from database inception through August 2025, using database-specific combinations of controlled vocabulary and free-text terms: (Woven EndoBridge OR WEB) AND ((“Stent-Assisted Coil” OR “Stent-Assisted Coiling” OR SAC) OR (“Microsurgical Clipping” OR “Surgical Clipping” OR MSC)) AND (Aneurysm OR Aneurysms) AND (“Wide-neck” OR “Wide-necked”). Following removal of duplicate records, titles and abstracts were independently screened by 2 reviewers (A.A. and J.F.O.C.), with subsequent full-text review of potentially eligible articles. Disagreements were adjudicated through discussion with a third reviewer (O.R.G.). Reference lists of included articles were hand-searched to identify additional relevant studies. Extracted data encompassed study-level characteristics (author, publication year, country, design, sample size, enrollment period), baseline patient demographics, aneurysm location and morphological features, procedural specifics (device generation and type, adjunctive techniques, stent type, antiplatelet protocol), and all outcomes of interest.

### 2.3. Outcomes

The primary efficacy outcome was adequate aneurysm occlusion at the last follow-up, as determined by angiography, according to Raymond–Roy classes I–II or corresponding classifications. The main safety endpoints were periprocedure thromboembolic or hemorrhagic complications and hemorrhage during the placement of the external ventricular drain (EVD). The secondary endpoints were complete occlusion of the aneurysm, recurrence or recanalization of the aneurysm, retreatment, favorable functional/neurological outcome, defined as a modified Rankin Scale (mRS) score of 0–2 at the final follow-up, procedure-related complications, technical success of the intervention, and time of the procedure or fluoroscopy time. The terms reported by original studies were left as they were; however, the outcomes across studies were harmonized whenever feasible for comparison in pooled analyses.

### 2.4. Quality assessment

Risk of bias in non-randomized studies was evaluated using the ROBINS-I tool,^[13]^ which assesses bias arising from confounding, participant selection, classification of interventions, deviation from intended interventions, missing outcome data, outcome measurement, and selective outcome reporting. Assessments were performed independently by 2 reviewers, and any discrepancies were resolved by consensus.

### 2.5. Statistical analysis

Pooled risk ratios (RR) with corresponding 95% confidence intervals (CIs) were calculated for dichotomous outcomes, while mean differences (MD) were used for continuous outcomes such as procedural time. Medians and interquartile ranges were converted to means and standard deviations where necessary.^[14,15]^ Random-effects models using restricted maximum likelihood (REML) estimation, with Hartung–Knapp adjustment, were applied throughout to account for between-study heterogeneity.

Statistical heterogeneity was assessed using the I^2^ statistic and Cochran’s Q test, with I^2^ thresholds of < 40%, 40–60%, and > 60% denoting low, moderate, and substantial heterogeneity, respectively. Prediction intervals were generated for the primary outcomes. To evaluate the robustness of the findings, sensitivity analyses excluded studies at high risk of bias and applied alternative statistical approaches for rare events (Peto method, generalized linear mixed models). Leave-one-out analyses were conducted by sequentially omitting individual studies to test the stability of pooled estimates, and publication bias was assessed using funnel plots and Egger’s test. Meta-regression was planned in the event that ≥ 10 studies reported a common outcome, to explore potential effect modifiers such as the proportion of ruptured aneurysms, dome-to-neck ratio, and mean patient age.^[16]^ All statistical analyses were conducted using R software (version 4.3.2), with statistical significance defined as a two-tailed *P* < .05.

## 3. Results

**Figure 1.**
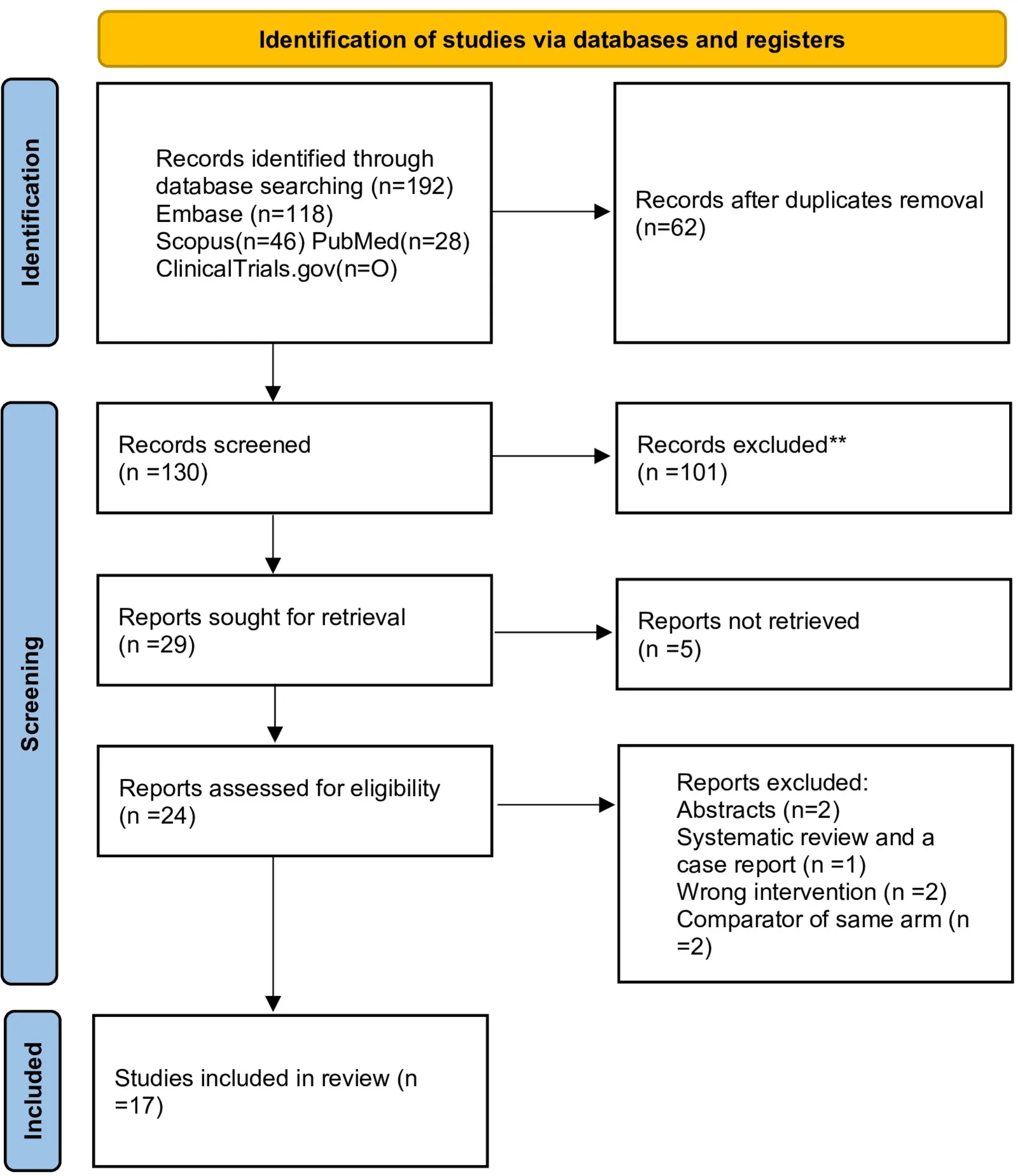
PRISMA flow.

A systematic search was performed from inception. A total of 192 records were retrieved: 118 from Embase, 46 from Scopus, 28 from PubMed, and zero from ClinicalTrials. The software identified 62 duplicate entries, which were removed prior to screening. A total of 130 records were initially screened, and 29 of them underwent full-text screening. Five studies were not retrieved due to a lack of available full text. During the full-text screening of 24 studies, two abstracts, two studies with incorrect interventions, one review, and two studies with incorrect controls were excluded. Finally, 17 papers were included in this review. Figure 1 shows the PRISMA flow diagram.

### 3.1. Baseline characteristics

A total of seventeen observational studies, encompassing 2289 patients, were included in our analysis. Three^[6,17,18]^ of these did not report the number of patients in each group. Among the remaining individuals, 701 (34.2%) were treated with WEB, 819 (40%) with SAC, and 531 (25.8%) with MSC. Information on patient age was missing in three^[6,17,18]^ studies, while three^[19–21]^ others presented age data as median values rather than means. Furthermore, ten articles^[6,11,17–19,22–25]^ omitted patients’ medical histories. One publication failed to indicate the number of unruptured and ruptured aneurysms.^[6]^ Regarding occlusion sites, a single study^[6]^ did not specify aneurysm location. Aneurysm size was unreported in two^[6,18]^ studies. Concerning neck width, four^[6,17,18,26]^ of the eighteen articles lacked this information. Finally, six^[6,11,18,24,26]^ studies did not provide the mean dome-to-neck ratio. Additional details on baseline characteristics are summarized in Table 1, and the pooled results of the meta-analysis are presented in Table 2.

**Table 1:** Baseline characteristics of the included studies.

Study (year)	T	M/F	Mean ± SD (or range) age, years	Comorbidities (N)	R/UN (N)	Aneurysm location (N)	Mean ± SD (or range) aneurysm size, mm	Mean ± SD (or range) neck width, mm	Mean ± SD (or range) dome-to-neck, mm
Baek (2024)	WEB	3/11	63.50 ± NR	NR	0/14	ICA (1) ACom (3) BA (4) MCA (6)	*4.79 (3.48–9.7)	*3.77 (2.7–6.89)	*1.28 (1.19–1.41)
	SAC	4/13	67.18 ± NR	NR	0/17	ICA (0) ACom (4) MCA (6) BA (7)	*4.84 (2.59–12.46)	*4 (2.27–9.37)	*1.14 (1.03–1.50)
Celik (2022)	WEB	9/40	61.9 ± 12.6	NR	0/40	BA (40)	6.2 ± 3.3	4.7 ± 1.4	1.4 ± 0.5
	SAC	6/29	63.2 ± 10.4	NR	0/35	BA (35)	6.2 ± 1.6	3.9 ± 1.7	1.7 ± 0.7
Chung (2024)	WEB	35/61	62.8 ± 9.6	HP (69)	0/96	ICA (6) BA (16) MCA (30) Acom (44)	5.4 ± 1.9	4 ± 1.4	1.4 ± 0.4
	SAC	67/123	61.5 ± 9.4	HP (119)	0/190	ICA (10) MCA (22) BA (56) ACom (102)	5.8 ± 2.4	4.1 ± 1.5	1.4 ± 0.3
El Naamani (2022)	WEB	22/41	62.4 ± 12.7	NR	16/47	ICA (4) BA (11) MCA (15) ACom (33)	5.9 ± 2.0	3.9 ± 1.4	NR
	SAC	26/59	60.2 ± 11.1	NR	15/70	ICA (6) MCA (10) ACom (14) BA (55)	7.2 ± 3.8	4.2 ± 1.7	NR
Ghanaati (2025)	WEB	10/5	56.53 ± 10.41	HP (9)	15/0	ICA (0) PICA (0) BA (3) MCA (4) ACA (8)	6.96 ± 1.43	4.35 ± 0.98	1.43 ± 0.17
	SAC	14/11	59.88 ± 10.16	HP (17)	25/0	PICA (1) BA (2) ACA (5) ICA (7) MCA (10)	5.68 ± 0.91	3.18 ± 0.52	1.45 ± 0.19
Goertz (2021)	WEB	23/40	65.2 ± 10.9	NR	0/63	MCA (18) ICA (19) ACom (26)	6.7 ± 2.2	3.8 ± 1.4	NR
	MSC	39/74	53.8 ± 10	NR	0/103	ICA (3) Acom (13) MCA (86)	6.6 ± 2.2	3.9 ± 1.5	NR
Goertz (2023)	WEB	NR***	NR†	NR	NR‡	NR§	NR§§	NR	NR
	SAC	NR***	NR†	NR	NR‡	NR§	NR§§	NR	NR
Hagen (2019)	WEB	13/25	59.8 ± 11.7	DM (2) HT (25)	0/38	MCA (38)	## 0–7 (19) 8–15 (19)	4.46 ± 1.26	2.55 ± 1.0
	SAC	17/50	55.7 ± 10.8	DM (7) HT (38)	0/67	MCA (67)	## 0–7 (47) 8–15 (16) 16–25 (4)	3.9 ± 1.5	2.60 ± 0.98
Hagen (2021)	WEB	5/16	53.6 ± 9.5	DM (0) HT (15)	21/0	MCA (21)	## 2–7 (14) 8–15 (7)	3.6 ± 0.8	2.7 ± 0.8
	SAC	9/26	53.9 ± 11.6	DM (6) HT (21)	35/0	MCA (35)	## 2–7 (21) 8–15 (10) 16–25 (1) > 25 (3	3.7 ± 1.4	3.5 ± 2.0
Kabbasch (2018)	WEB	21/45	57.5 ± 13.1	NR	24/42	MCA (9) ICA (13) ACom (16) BA (28)	7.3 ± 2.0	4.3 ± 1.2	1.6 ± 0.5
	SAC	15/51	55 ± 12.1	NR	24/42	MCA (9) ICA (13) ACom (16) BA (28)	7.2 ± 3.7	4.2 ± 2.0	1.6 ± -0.7
Kashkoush (2023)	WEB	7/28	*64 (50–70)	NR	0/35	ICA (3) BA (7) ACom (12) MCA (13)	*5.1 (4–6.3)	*3.2 (2.6–4.5)	*1.5 (1.2–1.8)
	SAC	19/51	*65 (57–69)	NR	0/70	ICA (4) MCA (19) BA (22) ACom (25)	*5.1 (3–6.5)	*4 (3–4.6)	*1.3 (1.1–1.7)
Park (2025)	WEB	12/25	62 ± 8.9	DM (10) HL (18) HP (21)	0/37	MCA (37)	4.94 ± 1.73	3.81 ± 1.31	1.32 ± 0.23
	MSC	65/186	60.1 ± 8.0	DM (51) HP (136) HL (146)	0/251	MCA (251)	4.75 ± 2.16	3.71 ± 1.41	1.29 ± 0.30
Rai (2021)	WEB	NR	NR	NR	13/33	ICA (1) Pcom (3) BA (9) MCA (14) ACom (19)	5.3 ± 1.7	NR	1.5 ± 0.3
	SAC	NR	NR	NR	4/37	ICA (0) MCA (6) PCom (7) ACom (8) BA (20)	5.9 ± 1.9	NR	1.5 ± 0.3
Rodriguez-Calienes, 2024	WEB	44/61	*56 (48–64)	DM (19) HL (28) HP (55)	105/0	ICA (7) PCA (9) Other (10) BA (11) MCA (30) ACom (38)	*6.3 (5–8)	*4 (3–5)	*1.5 (1.3–1.7)
	SAC	42/80	*56 (48–69)	DM (11) HL (25) HP (58)	112/0	BA (0) PCA (0) MCA (10) Other (29) ACA (35) ACom (38)	*4.6 (3.2–6.2)	*3.3 (2.5–4.3)	*1.5 (1.2–1.7)
Roy (2025)	WEB	24/37	*73 (61.5–79.5)	AF (7) PS (11) DM (12) CAD (17) HL (32) HP (50)	15/46	ICA (7) MCA (13) PC (15) ACA (26)	*5.7 (4.2–7.6)	*4.4 (3.1–5.3)	*1.3 (1–1.7)
	MSC	94/33	*56 (48–65)	CAD (4) AF (5) DM (16) PS (18) HL (42) HP (83)	27/100	MCA (1) PC (1) ICA (54) ACA (71)	*4.4 (3.2–6.3)	*3.1 (2.4–4.1)	*1.8 (1–1.8)
Styczen (2021)	WEB	**NR	‖‖NR	NR	1/5	Mid-BA (1) Proximal BA (5)	¶NR	NR	#NR
	SAC	**NR	‖‖NR	NR	4/3	Mid-BA (1) Proximal BA (6)	¶NR	NR	#NR
Sauvigny (2019)	WEB	12/26	56.7 ± 13.7 (29–86)	CD (1) ND (4) HP (17)	38/0	MCA (3) PC (5) ICA (8) ACA (22)	6.6 ± 2.9 (1–16)	NR	NR
	MSC	25/31	56.1 ± 12 (35–80)	ND (4) CD (6) HP (22)	56/0	PC (0) ICA (12) ACA (13) MCA (31)	6.2 ± 3.6 (1–18)	NR	NR
	SAC	37/70	54.8 ± 13.8 (21–90)	ND (11) CD (18) HP (51)	107/0	MCA (4) PC (16) ICA (29) MCA (58)	6.8 ± 4.9 (1.3–26)	NR	NR

ACA = anterior cerebral artery, ACom = anterior communicating artery, AF = atrial fibrillation, BA = basilar artery, CAD = coronary artery disease, CD = cardiovascular disease, DM = diabetes mellitus, F = female, HL = hyperlipidemia, HP = hypertension, ICA = internal carotid artery, M = male, MCA = middle cerebral artery, mm = millimiter, MSC = microsurgical clipping, NR = not reported, PC = posterior circulation, PCA = posterior cerebral artery, PCom = posterior communicating artery, PICA = posterior inferior cerebellar artery, PS = previous stroke, R = ruptured aneurysm, SAC = stent coiling assisted, T = treatment, UN = unruptured aneurysm.

* Median (IQR) instead of mean.

** The study did not specify the sex distribution within the WEB and SAC groups. However, it reported a total of 8 men and 27 women.

*** The study did not specify the sex distribution of the WEB and SAC groups. However, it provided a total of 33 men and 67 women.

† Although the study did not report the mean age separately for the WEB and SAC groups, it provided an overall mean age of 55 years (SD: 10.2).

‡ Despite not specifying the number of unruptured and ruptured aneurysms within the WEB and SAC groups, the study reported a total of 100 unruptured aneurysms.

§ The study did not provide data regarding aneurysm location in the WEB and SAC groups separately. However, all included aneurysms were located in the MCA.

§§ Although the study did not report aneurysm size separately for the WEB and SAC groups, the reported diameters ranged from 3 to 11 mm.

‖‖ The study did not specify the mean age of the WEB and SAC groups. However, it provided a median age of 55 years (range: 23–79).

¶ Although the study did not report aneurysm size separately for the WEB and SAC groups, it provided an overall mean size of 7.0 ± 4.9 mm.

# Although the study did not report dome-neck ratio separately for the WEB and SAC groups, it provided an overall ratio of 1.3 ± 0.7 mm.

## The study provided size ranges and their respective counts, instead of a mean value.

**Table 2:** Summary of the pooled findings.

		Group	Events	Total	Risk ratio M-H, random (95% CI)	Heterogeneity, I^2^	*P*-value
WEB vs MSC							
**Outcomes**	Adequate occlusion	WEB	103	146	0.77 (0.69–0.86)	0.0%	<.001
		MSC	421	448			
	Incomplete occlusion	WEB	43	146	4.61 (2.12–10.01)	63.1%	<.001
		MSC	27	448			
	Mortality	WEB	6	161	3.25 (0.96–10.94)	0.0%	.057
		MSC	4	481			
	mRS (0–2)	WEB	167	192	0.99 (0.96–1.02)	0.0%	.433
		MSC	494	532			

WEB vs SAC							
**Outcomes**	Complete occlusion	WEB	304	421	0.98 (0.90–1.07)	47.9%	.617
		SAC	350	496			
	Retreatment	WEB	26	412	0.79 (0.39–1.59)	42.1%	.508
		SAC	43	503			
	mRS (0–2)	WEB	379	427	1.03 (0.99–1.07)	22.9%	.141
		SAC	409	488			
	Thromboembolic complications	WEB	11	140	0.59 (0.29–1.18)	0.0%	.137
		SAC	19	140			
	Procedure-related complications	WEB	17	338	0.49 (0.29–0.85)	0.0%	.011
		SAC	41	395			

MSC = microsurgical clipping, SAC = stent-assisted coiling, WEB = Woven EndoBridge.

### 3.2. WEB vs MSC

#### 3.2.1. Adequate occlusion

**Figure 2.**
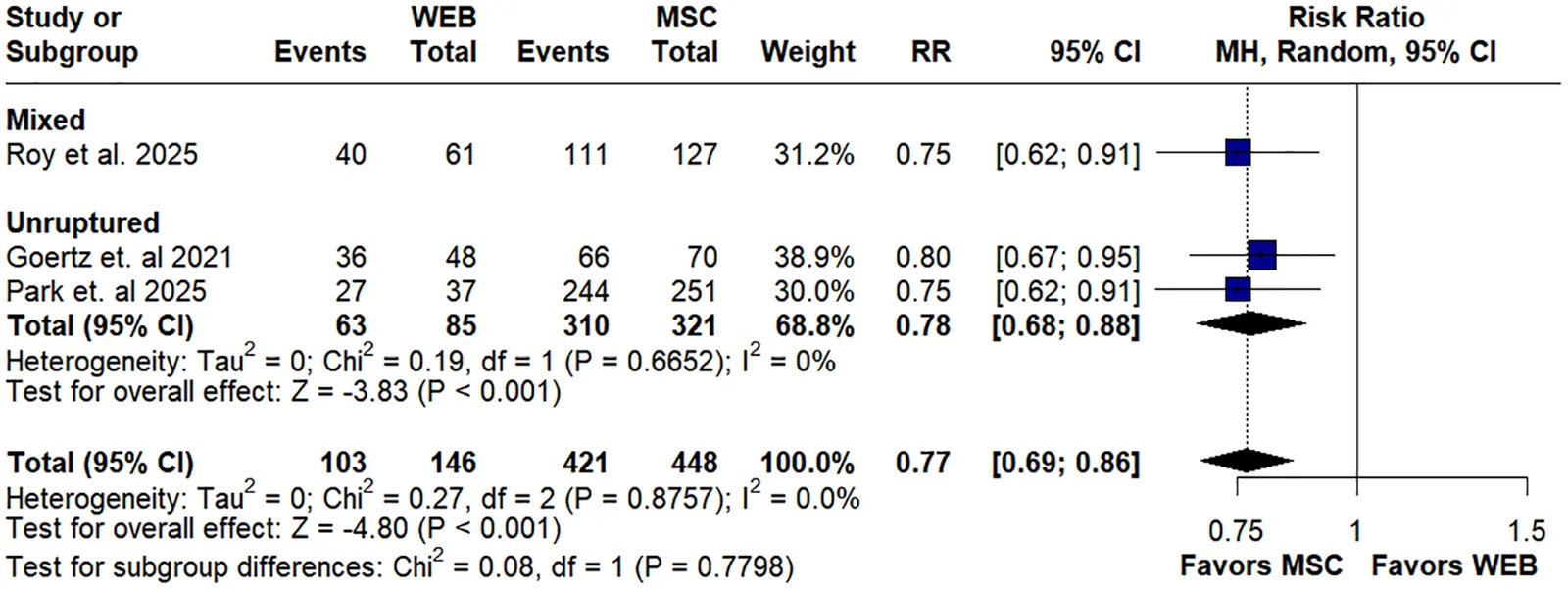
Forest plot for adequate occlusion.

Incidence of adequate occlusion was significantly lower in the WEB group (RR 0.77; 95% CI 0.69–0.86; *P* < .001; I^2^ = 0.0%; Fig. 2). Mixed and unruptured aneurysm subgroups yielded significant results, but there was no significant difference between subgroups (*P* = .78).

#### 3.2.2. Incomplete occlusion

**Figure 3.**
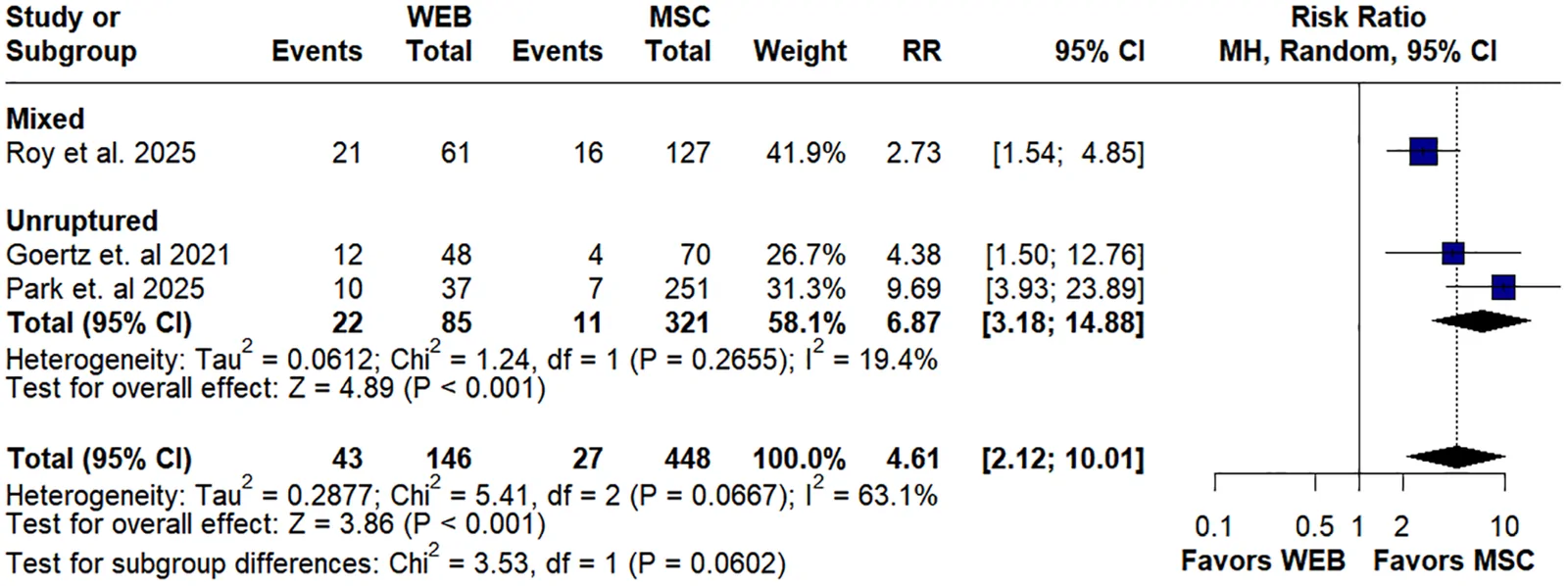
Forest plot for incomplete occlusion.

The incidence of incomplete occlusion occurred significantly more often in the WEB group (RR 4.61; 95% CI 2.12–10.01; *P* < .001; I^2^ = 63.1%; Fig. 3). Both mixed and unruptured subgroups showed a significantly lower incidence of incomplete occlusion in MSC group. There was no significant difference between mixed and unruptured subgroups (*P* = .06). Exclusion of Park 2025 reduced I^2^ to 0%, and results remained significant (RR 3.04; 95% CI 1.83–5.04).

#### 3.2.3. Mortality

**Figure 4.**
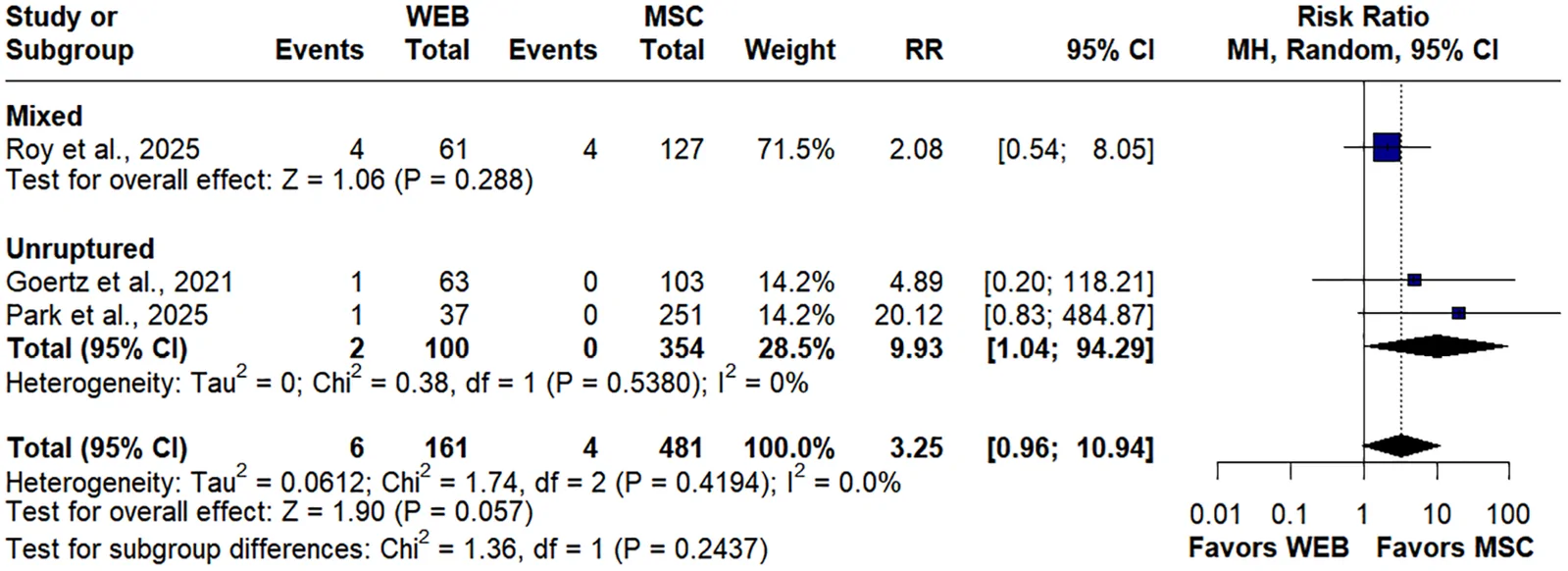
Forest plot for mortality.

There was no significant difference in mortality (RR 3.25; 95% CI 0.96–10.94; *P* = .057; I^2^ = 0.0%; Fig. 4). There was no significant difference between mixed and unruptured subgroups (*P* = .24), and both subgroups yielded insignificant results.

#### 3.2.4. Favorable neurological outcome

**Figure 5.**
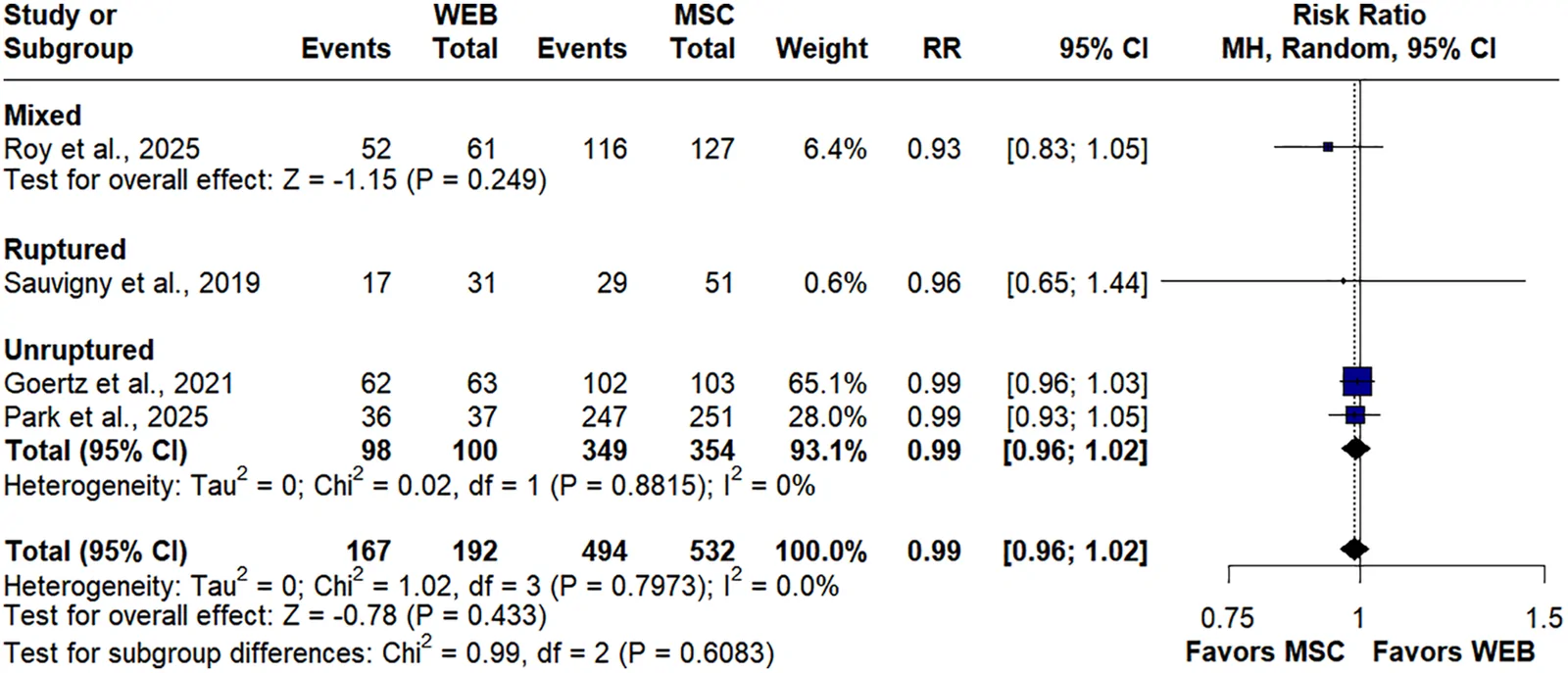
Forest plot for favorable neurological outcome.

There was no significant difference in the proportion of patients achieving favorable neurological outcome between the 2 groups (RR 0.99; 95% CI 0.96–1.02; *P* = .433; I^2^ = 0.0%; Fig. 5). There was no significant difference between mixed, ruptured, and unruptured aneurysm subgroups (*P* = .61), and all 3 subgroups yielded insignificant results.

### 3.3. WEB vs SAC

#### 3.3.1. Complete occlusion

**Figure 6.**
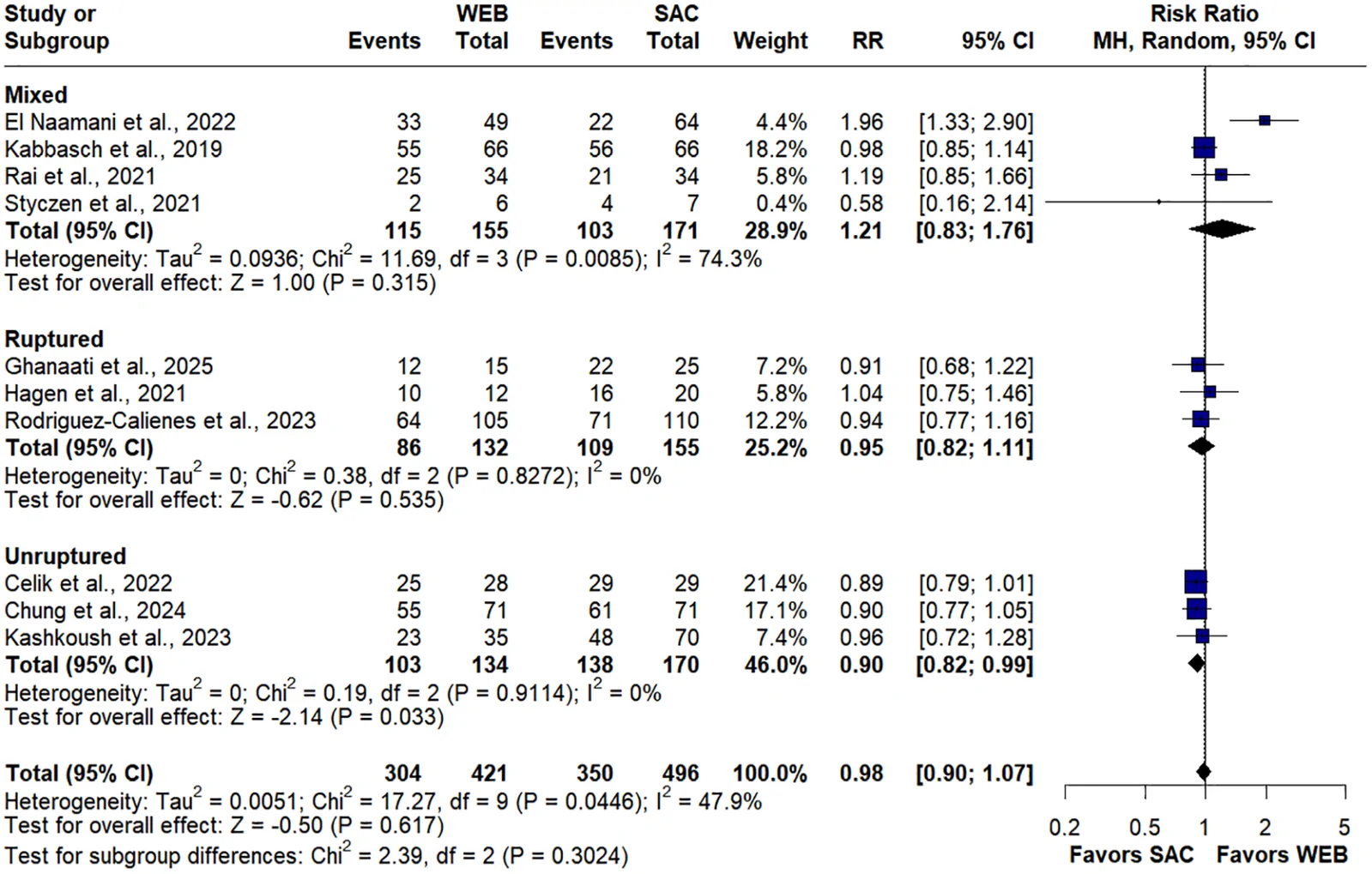
Forest plot for complete occlusion.

There was no significant difference in the number of patients with complete occlusion (RR 0.98; 95% CI 0.90–1.07; *P* = .617; I^2^ = 47.9%; Fig. 6). There was no significant difference between ruptured, unruptured, and mixed aneurysm subgroups (*P* = .30), and all subgroups yielded insignificant results. Exclusion of El Naamani (2022) reduced the I^2^ to 0%, but results remained insignificant. Visual assessment of the funnel plot showed no publication bias, and this was confirmed through Egger’s test (*P* = .23).

#### 3.3.2. Retreatment

**Figure 7.**
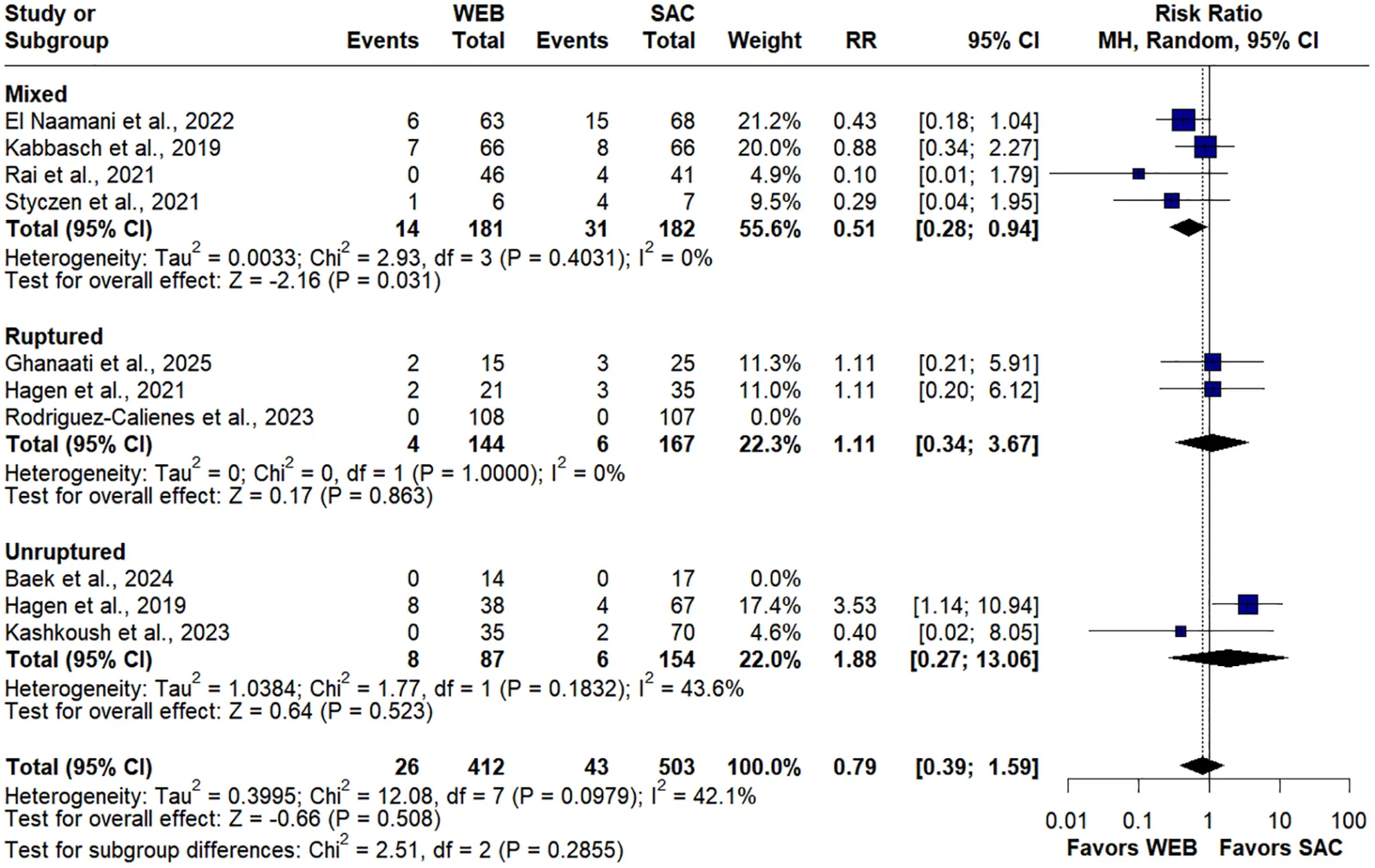
Forest plot for retreatment.

There was also no significant difference in retreatment between the 2 groups (RR 0.79; 95% CI 0.39–1.59; *P* = .508; I^2^ = 42.1%; Fig. 7). Only mixed aneurysm subgroup yielded significant results (RR 0.51; 95% CI 0.28–0.94; *P* = .031; I^2^ = 0.0%); however, there was no significant difference between the subgroups (*P* = .29). After excluding Hagen (2019) study, I^2^ reduced to 0%, but results remained insignificant. Similarly, no publication bias was observed (*P* = .54).

#### 3.3.3. Favorable neurological outcome

**Figure 8.**
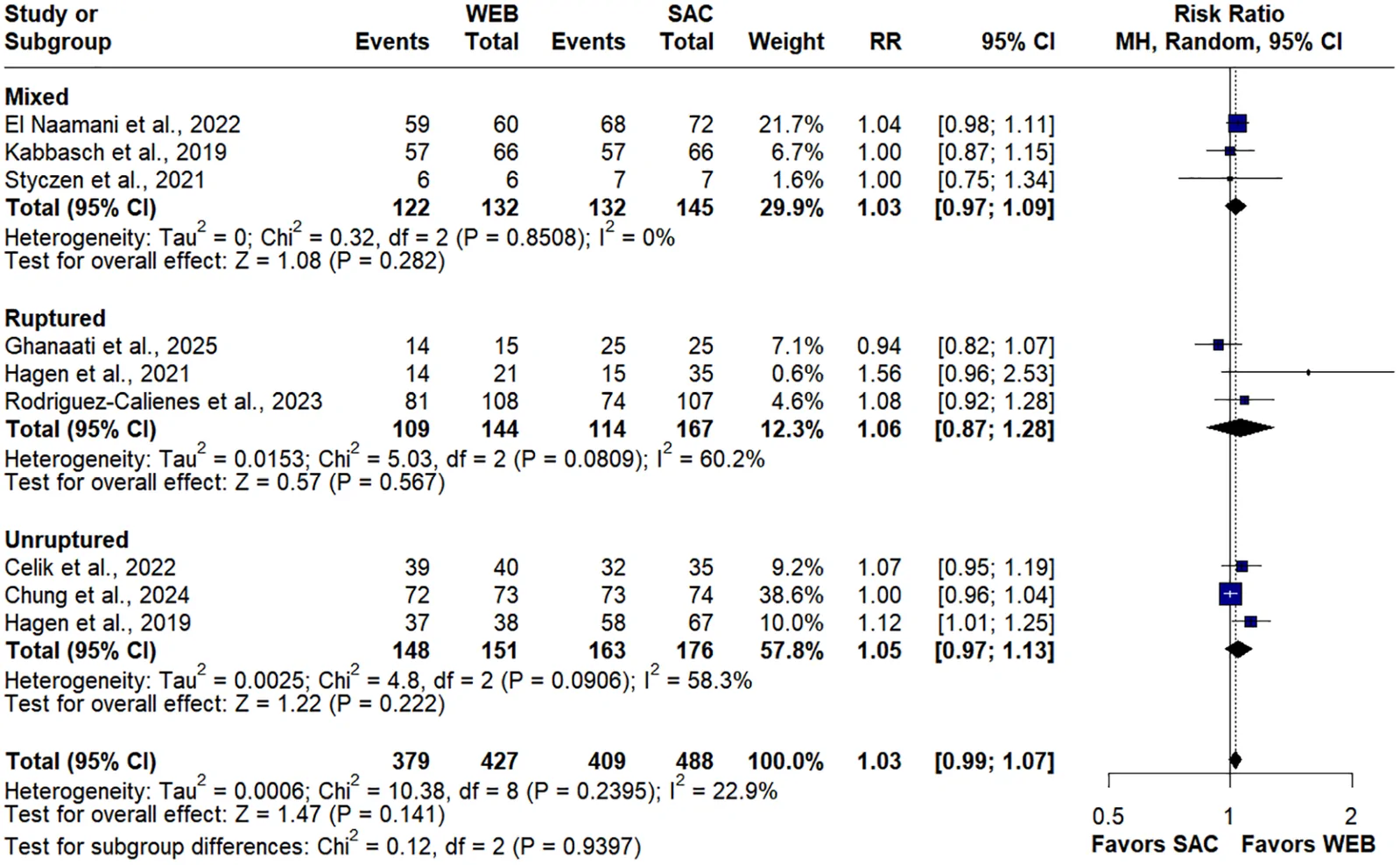
Forest plot for favorable neurological outcome.

There was no significant difference in the proportion of patients with favorable neurological outcomes (RR 1.03; 95% CI 0.99–1.07; *P* = .141; I^2^ = 22.9%; Fig. 8). All subgroups yielded insignificant results and there was no significant difference between them (*P* = .94). In LOO analysis, exclusion of studies Chung (2022) or Ghanaati (2025) yielded significant results: RR 1.04; 95% CI 1.00–1.08; I^2^ = 19% after exclusion of Chung (2022), and RR 1.05; 95% CI 1.00–1.09; I^2^ = 12%, after exclusion of Ghanaati (2025), favoring WEB group, Publication bias test showed insignificant results (*P* = .18).

#### 3.3.4. Thromboembolic complications

**Figure 9.**
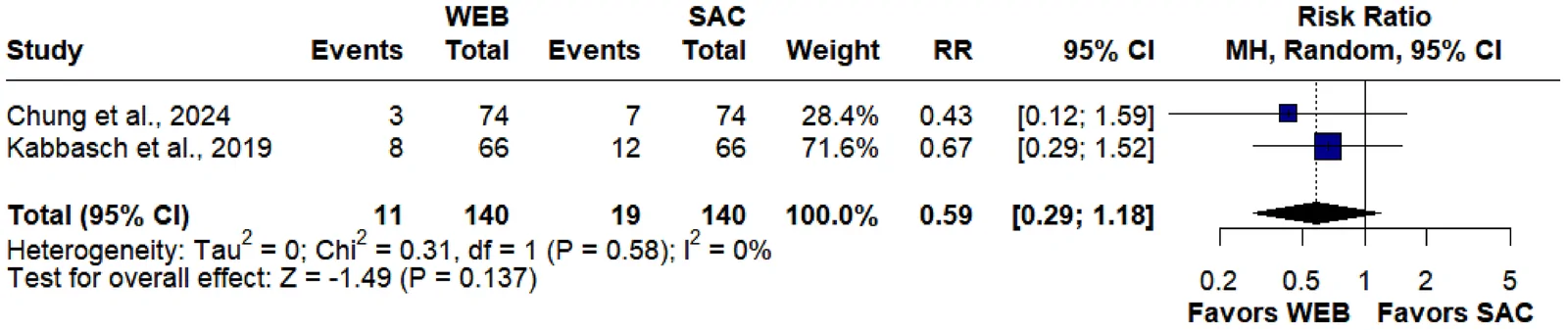
Forest plot for thromboembolic complications.

There was no significant difference in thromboembolic complications between WEB and SAC groups (RR 0.59; 95% CI 0.29–1.18; *P* = .137; I^2^ = 0.0%; Fig. 9).

#### 3.3.5. Procedure-related complications

**Figure 10.**
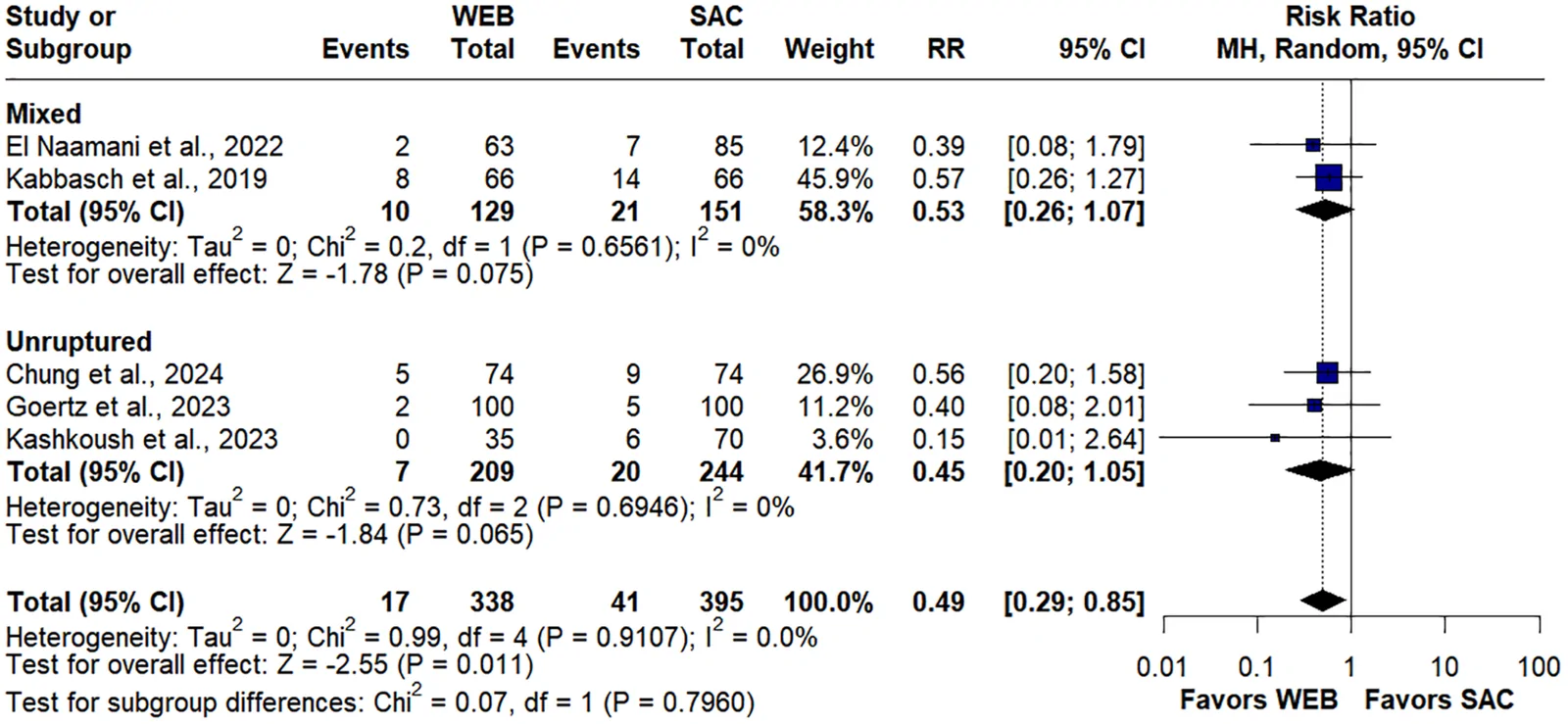
Forest plot for complications.

Incidence of procedure-related complications was significantly reduced in the WEB group (RR 0.49; 95% CI 0.29–0.85) *P* = .011; I^2^ = 0.0%; Fig. 10). Both mixed and unruptured subgroups yielded insignificant results and there was no difference between them (*P* = .796).

### 3.4. Quality assessment

**Figure 11.**
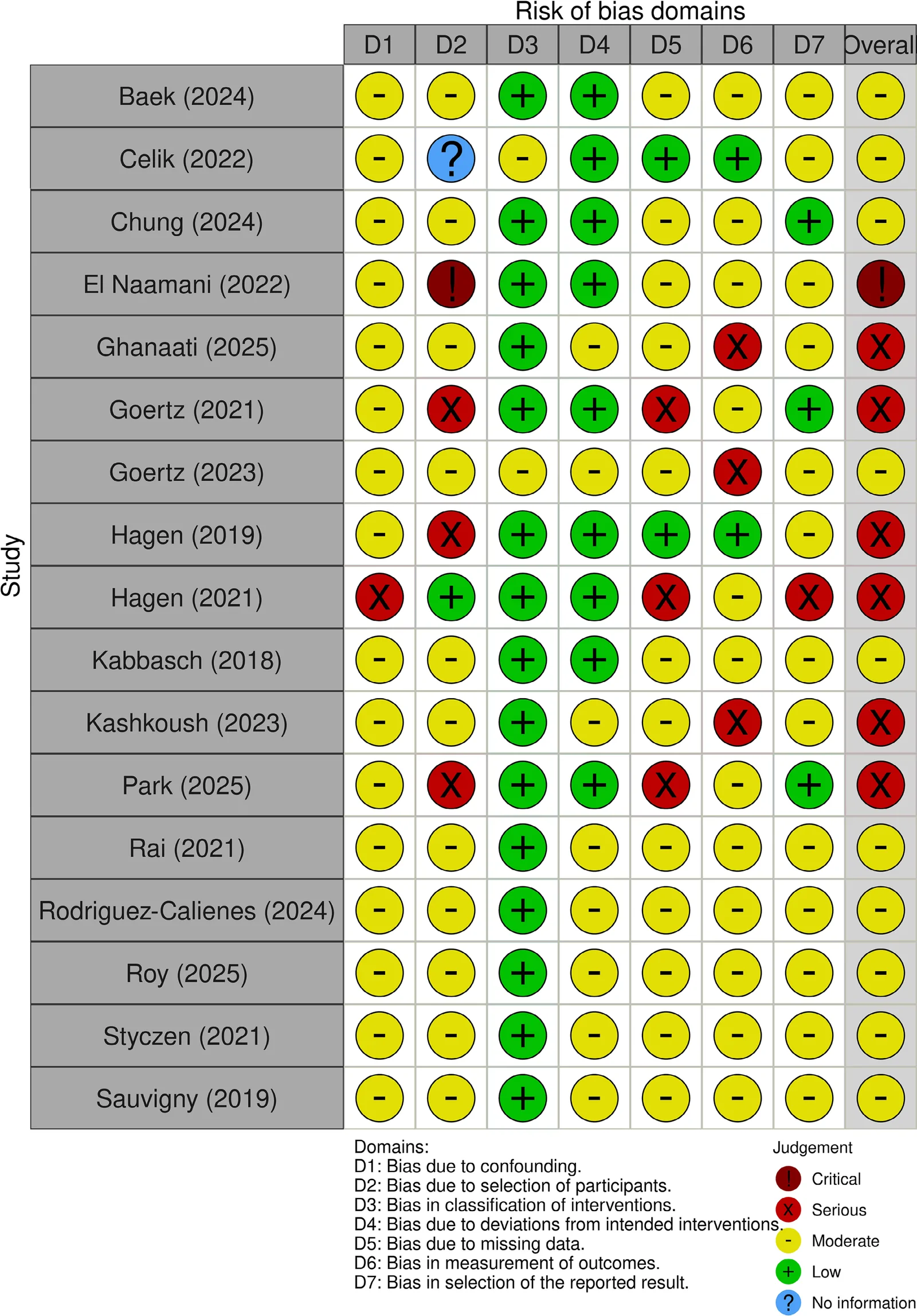
Risk of bias, ROBINS-I.

As seen in Figure 11, none of the included studies had a low risk of bias across all ROBINS-I domains. Three studies (Celik, 2022; Rai, 2021; Rodriguez-Calienes, 2024) were rated as having an overall moderate risk, with no domains judged serious or critical. Most studies had at least one domain with a serious risk, most often D2 (confounding) or D6 (selection of the reported result). Two studies (El Naamani, 2022; Park, 2025) had a critical overall risk, reflecting serious or critical concerns in multiple domains.

## 4. Discussion

This systematic review and meta-analysis evaluated the efficacy and safety of the WEB device against stent-assisted coiling (SAC) and simple coiling (MSC) for treating ruptured and unruptured intracranial aneurysms. Compared with MSC, WEB showed a lower rate of complete occlusion, and a higher rate of incomplete occlusion. However there were no differences with mortality or neurological outcome. WEB compared to SAC showed similar results in complete occlusion, re-treatment, thromboembolic complications, and neurological outcome; except for procedure-related complication rates. This finding brings us to a point where there is a distinct clinical decision to be made; treatment using clipping would provide long-term patency but WEB may be a better choice in patients where safety and ease of procedure is paramount, therefore choice of treatment method should be individual based and taken from aneurysm features and individual morbidities along with institutional expertise.

The WEB device was developed as an intrasaccular flow disruption technology designed to exclude the aneurysm from normal circulation. Attainment of ~35–45% of metal coverage across the neck limits blood flow across the lesion and hence favors the formation of thrombus within the lumen while at the same time acting as scaffold for neointimal proliferation.^[27]^ The WEB design, with braided structure and self-expansion, guarantees radial force that allows precise and stable anchoring and also shapes in the most irregular aneurysm anatomy, including wider neck diameters and complex geometries^.[28]^ Clinical evidence from the WEBCAST, WEBCAST-2, and French Observatory trials has demonstrated complete occlusion rates around 52.9%, with neck remnants observed in 26.1% of cases.^[4,29–31]^ Moreover, a large multicenter analysis, which included 206 middle cerebral artery bifurcation aneurysms, reported adequate occlusion in 78.6% of patients.^[32]^ Although these single-arm studies suggest encouraging efficacy and safety, comparative data between WEB and established treatment modalities remain limited.

In relation to microsurgery clipping, the rates of adequate occlusion were worse with the WEB device with increase in rates of incomplete occlusion, which demonstrates in an efficient way the better durability of the clipping to achieve exclusion of the aneurysm for long periods. This is consistent with micresurgical intervention as the gold standard in the treatment of the intracranial aneurysms, and particularly in young patients whose conditions may allow for surgery. On the other hand, Clipping is highly invasive with the risk of perioperative complication in the elderly patients and patients with comorbidities, where the WEB device is potentially a tool of risk avoidance for those patient, whereas results in long term follow up can be in favor to the clipping in what refer to occlusions rates. Differences in rates between clipping and WEB device have no clearly and visibly translated to differences inmortality or functional outcomes, meaning differences on radiographic imaging dont necessarily translate in clinically visible differences.

High morbidity rates have traditionally been cited as a limitation of craniotomy.^[33]^ Nevertheless, a recent meta-analysis by Algra et al^[34]^ reported that clipping of unruptured aneurysms was associated with an 8% complication rate and a remarkably low mortality of 0.1%. When considering the WEB device, mid-term morbidity appears comparable to that observed with conventional coiling.^[25,35]^ Reported mortality related to WEB use is mainly restricted to ruptured aneurysms. Lawson et al^[36]^ described mortality in 5 patients with unruptured basilar artery aneurysms; however, these deaths were not attributable to the procedure itself. Goertz et al^[11]^ reported that unfavorable outcomes at 6 months occurred in <2% of patients treated with either WEB devices or microsurgical clipping, suggesting comparable safety between the 2 methods for the analyzed aneurysm subset.

Concerning functional outcomes assessed by the mRS score, craniotomy and subarachnoid dissection may contribute to postoperative cognitive deficits, anxiety, and depression.^[37]^ On the other hand, long-term angiographic follow-up following endovascular treatment, with the potential need for retreatment in cases of incomplete occlusion or recanalization, can also trigger anxiety and depressive symptoms.^[38]^ Given its favorable safety and efficacy, clipping is expected to continue playing an important role in the management of anterior circulation aneurysms, particularly for large or complex lobulated aneurysms that are not ideal candidates for WEB treatment.^[39,40]^

In our study, in the comparison with stent-assisted coiling, the WEB demonstrated largely equivalent efficacy across angiographic and clinical endpoints, including occlusion rates, retreatment, and thromboembolic complications. Importantly, however, the WEB was associated with a lower incidence of procedure-related complications, suggesting a perioperative safety advantage. The observed difference might stem from the technical straightforwardness of WEB deployment, which bypasses the requirement for dual antiplatelet therapy and mitigates risks associated with stent placement. Clinically, these results suggest WEB is a viable alternative to SAC, especially for ruptured aneurysms or in patients for whom extended antiplatelet therapy is contraindicated. Nevertheless, with comparable efficacy, the selection between these endovascular methods should account for operator experience, device accessibility, and patient-specific anatomy. Like WEB, SAC is principally employed to embolize wide-neck aneurysms and avert coil protrusion into the parent vessel. Research indicates SAC generally offers more lasting aneurysm occlusion than coiling alone.^[41–43]^ In a large cohort of 888 aneurysms, Piotin et al reported a recurrence rate of 14.9% following SAC versus 33.5% after standalone coiling.^[44]^ Similarly, Jahshan et al observed complete occlusion in 64.6% of aneurysms treated with SAC compared to 49.7% with coiling alone in a series of 489 aneurysms.^[43]^ Kabbasch et al^[25]^ reported analogous findings, achieving complete occlusion in 84.8% and adequate occlusion in 93.9% of cases. These data suggest that for wide-neck and bifurcation aneurysms, treatment with the WEB device yields occlusion rates comparable to those with the SAC.

In summary, for intracranial aneurysms, the main trade-off in choosing a treatment strategy appears to be between the durability of occlusion and procedural safety. While MSC may still be the gold standard for angiographic durability, its inherent invasive risks likely restrict its usage to younger, healthier individuals with anatomically favorable lesions. SAC and WEB both offer equally comparable alternatives with comparable safety profiles at present and WEB may also pose an additional benefit with reduced perioperative complications and without a need for dual antiplatelet therapy. These data reinforce a patient and lesion-specific treatment approach, where the geometry of the aneurysm, ruptured status, underlying patient conditions, and expertise of the institution dictate what the best modality of therapy is.

Further high-quality randomized controlled trials directly comparing WEB, SAC, and MSC are needed to better define the optimal treatment strategy for different patient subgroups. Further long-term follow-up is essential, particularly regarding the durability of WEB occlusion and the likelihood of aneurysm recurrence or retreatment. Technological improvements in the device along with an increasing operator experience may enhance the angiographic outcome with WEB in the future. Cost-effectiveness analysis may also be pertinent for healthcare system resource allocation.

### 4.1. Limitations

The limits of this review stem largely from the heterogeneity among the studies involved. Areas of variation among studies included: definition of the patient population, type of aneurysms being studied, and duration of follow-up time periods. Most of the data included comes from observational studies which are prone to inherent flaws in patient selection, and confounding variables. The data that exists is from a small number of studies that compare the WEB device to mechanical self-expanding coils (MSC) limiting strength of conclusions to operative versus endovascular treatment. Additionally, there was heterogeneity in the reporting of outcomes and complications in individual studies which influenced pooled estimates.

## 5. Conclusion

Microsurgical clipping appears to offer the most durable form of aneurysm occlusion. In contrast, the WEB device and self-expanding aneurysm coils (SAC) represent less invasive options that demonstrate generally comparable efficacy. The WEB device exhibits a more favorable safety profile relative to SAC, although its angiographic durability is lower than that achieved with microsurgical clipping (MSC). Consequently, the selection of treatment should be tailored to the individual, taking into account patient-specific factors, the morphology of the aneurysm, and the expertise available at the treating institution.

## Author contributions

**Conceptualization:** Adil Ahmed, Ocílio Ribeiro Gonçalves, Muhammad Nawaz Khan, Syed Sarmad Bukhari.

**Formal analysis:** Adil Ahmed.

**Investigation:** Adil Ahmed.

**Methodology:** Adil Ahmed, Maria Antonia Oliveira Machado Pereira.

**Project administration:** Adil Ahmed.

**Resources:** Adil Ahmed.

**Software:** Adil Ahmed, Kenzo Ogasawara Donato.

**Supervision:** Adil Ahmed.

**Visualization:** Adil Ahmed.

**Writing – original draft:** Adil Ahmed, Ocílio Ribeiro Gonçalves, Paweł Łajczak, Kenzo Ogasawara Donato, Jose Fernando Olvera-Castro, João Vitor Andrade Fernandes, Syed Sarmad Bukhari.

**Writing – review & editing:** Adil Ahmed, Ocílio Ribeiro Gonçalves, Paweł Łajczak, Filipe Virgilio Ribeiro, Anderson Matheus Pereira da Silva, João Vitor Andrade Fernandes, Muhammad Nawaz Khan, Syed Sarmad Bukhari.

**Data curation:** Jose Fernando Olvera-Castro.
